# Dispositional self‐compassion and responses to mood challenge in people at risk for depressive relapse/recurrence

**DOI:** 10.1002/cpp.2302

**Published:** 2018-06-12

**Authors:** Anke Karl, Matthew J. Williams, Jessica Cardy, Willem Kuyken, Catherine Crane

**Affiliations:** ^1^ Mood Disorders Centre University of Exeter UK; ^2^ Oxford Doctoral Course in Clinical Psychology, Harris Manchester College University of Oxford Oxford UK; ^3^ Department of Psychiatry University of Oxford Oxford UK

**Keywords:** compassion, depression, emotion regulation, mindfulness, mood induction, self‐compassion

## Abstract

This paper explores the relationship between dispositional self‐compassion and cognitive emotion regulation capacities in individuals with a history of depression. Study 1 (*n* = 403) established that self‐compassion was associated with increased use of positive and decreased use of negative strategies, with small to medium sized correlations. Study 2 (*n* = 68) was an experimental study examining the association between dispositional self‐compassion, use of cognitive emotion regulation strategies, and changes in mood and self‐devaluation in participants exposed to a negative mood induction followed by mood repair (mindfulness, rumination, silence). Individuals with higher levels of dispositional self‐compassion showed greater mood recovery after mood induction, and less self‐devaluation across the experimental procedure, independent of their mood‐repair condition or habitual forms of cognitive emotion regulation. These results suggest that self‐compassion is associated with more adaptive responses to mood challenges in individuals with a history of recurrent depression.

**Key Practitioner Message:**

- Higher levels of dispositional self‐compassion are associated with selection of more adaptive cognitive emotion regulation strategies in people with a history of depressive relapse/recurrence.
- Dispositional self‐compassion is also associated with greater mood repair following a mood challenge.

## 1 INTRODUCTION

Depression is a prevalent disorder associated with significant impairment and suffering (Collins et al., 2011). It typically runs a recurrent course, with rates of recurrence/relapse greater than 50% after a first episode and 90% for those who have experienced three or more episodes (Solomon et al., 2000). To develop and refine psychological interventions to support sustained recovery from depression, it is essential to have a clear understanding of the mechanisms involved in the processes of relapse and recurrence (Clark, 2004).

The Differential Activation Theory of depressive relapse/recurrence (Segal, Williams, Teasdale, & Gemar, 1996) proposes that in people at risk of depressive relapse/recurrence, sad mood becomes associated with negative beliefs, higher order meanings, and a tendency to ruminate. As a consequence, brief periods of low mood are thought to automatically trigger negative content, for example, negative self‐devaluative thinking (Scher, Ingram, & Segal, 2005). Subsequently, or in parallel, a range of maladaptive cognitive processes (such as biases in memory and interpretation, e.g., J. M. G. Williams, Watts, MacLeod, & Mathews, 1997), and deficits in behavioural functioning (such as impaired interpersonal problem solving) occur (e.g., J. M. G. Williams, Barnhofer, Crane, & Beck, 2005), which together exacerbate and prolong low mood, may also be triggered, increasing risk of escalation into a depressive episode (Beck & Haigh, 2014; Teasdale & Barnard, 1993; Teasdale & Cox, 2001). Indeed, recent research demonstrates that mood‐induced activation of depressogenic cognitions, and in particular rumination, is a better predictor of relapse to depression over a 3.5‐year period than the level of these cognitions in euthymic mood (e.g., Figueroa et al., 2015). These findings suggest that, in order to avoid depressive relapse in response to transient low mood, individuals with a history of depression need to apply skills and resources to respond to this mood and the negative thought content that might automatically and habitually be triggered in a more adaptive way and negative events in people with a history of depression, and the way these responses relate to individual differences in dispositional self‐compassion.

Compassion has been defined as “an orientation of mind that recognizes the presence of pain, the universality of pain in human experience and the capacity to meet pain with kindness, empathy, equanimity and patience” (Feldman & Kuyken, 2011). *Self*‐compassion refers to the ability to relate to one's own experience with the same qualities of mind and has been described as comprising three core dimensions: self‐kindness versus self‐judgement, common humanity versus isolation, and mindfulness versus over‐identification (Neff, 2003b). The relationship between mindfulness and self‐compassion is complex. Several of the key elements of self‐compassion (recognizing and attending to suffering, being able to be open to and tolerate these thoughts and feelings, alongside an attitude of care) are present in definitions of mindfulness. Likewise, the ability to attend to and hold pain in awareness seen as a necessary prerequisite for self‐compassion (Neff, 2003a, 2003b). However, the important elements of common humanity and motivation to act/acting to alleviate suffering (Strauss et al., 2016) are distinct to the concept of self‐compassion. In this paper, we use the term dispositional self‐compassion to refer to people's self‐reported general tendency to be compassionate towards themselves.

It is suggested that when a person at risk for depressive relapse responds to negative mood with self‐compassion, the subsequent prolongation or exacerbation of low mood may be diminished (Krieger, Berger, & Holtforth, 2016; Pauley & McPherson, 2010) because the negative thoughts and feelings that arise can more easily be seen for what they are, over‐learned and unhelpful beliefs and attitudes. Rather than engaging in increasingly negative, self‐critical and self‐devaluative thinking, when a person is able to observe their thoughts and feelings compassionately, contextualizing them not only within their own personal life story or narrative but also within the context of broader human experience, this may reduce avoidance of negative mental content and facilitate adaptive responding, including seeing things from a new perspective, engaging in positive reappraisal and making positive behavioural change. Self‐compassion is potentially important for people vulnerable to depressive relapse because it can be invoked at the times of greatest risk for depression (e.g., low mood and cognitive reactivity), bypasses self‐devaluative processing (e.g., self‐criticism, catastrophizing, rumination) and may enable people to step out of reactivity and utilize resilient cognitive and behavioural strategies.

There are now a number of correlational studies showing a relationship between self‐compassion, well‐being, and mental health (Broderick, 2005; Fredrickson, Cohn, Coffey, Pek, & Finkel, 2008; Huffziger & Kuehner, 2009; Krieger et al., 2016; Neff, Rude, & Kirkpatrick, 2007). Studies show that self‐compassion is consistently negatively associated with avoidance, rumination, and depressive symptom severity in depressed patients (e.g., Krieger, Altenstein, Baettig, Doerig, & Holtforth, 2013), that levels of self‐compassion and self‐criticism differentiate currently and previously depressed individuals from never‐depressed controls (e.g., Ehret, Joorman, & Berking, 2015) and that the association between self‐compassion and depressive symptoms is mediated by the ability to tolerate negative emotions, but not other emotion regulation skills (Diedrich, Burger, Kirchner, & Berking, 2017). Interestingly, a review of the literature on the association between self‐compassion and coping strategy use by Allen and Leary (2010) suggests that the association between self‐compassion and the use of positive cognitive restructuring/reappraisal is most evident, consistent with the idea that effective regulation of emotional states, including through self‐compassion, limits escalation of negative affect and facilitates subsequent engagement in positive coping responses.

Experimental studies have also examined the association between self‐compassion and emotional responses to stress or induced negative mood. For example, across a series of five studies with non‐clinical, largely undergraduate student samples, Leary, Tate, Adams, Allen, and Hancock (2007) demonstrated that self‐compassion attenuated emotional reactions to a range of stressful real, remembered, and imagined events. Such findings are consistent with another recent study examining the naturalistic relationship between self‐compassion, affect and daily stressors in 101 participants who provided mood and stressor data twice daily (Krieger, Hermann, Zimmermann, & Holtforth, 2015). This study showed that self‐compassion attenuated the effect of daily stressors on negative affect, although not positive affect, over a 2‐week period.

A majority of studies of self‐compassion and emotion regulation to date have relied on non‐clinical samples. However, one recent experimental study of 48 people meeting criteria for depression compared self‐compassion with a range of other emotion regulation strategies intended to repair mood following a sad mood induction (Diedrich, Grant, Hofmann, Hiller, & Berking, 2014). This study suggested that deliberately activating feelings of self‐compassion (i.e., using self‐compassion as a form of emotion regulation) was more effective than a simple waiting period in reducing depressed mood following a mood induction but did not differ significantly from either acceptance or reappraisal. In a second study, conducted concurrently with the work reported here, this group considered the relationship between emotion regulation and the ability of depressed patients to benefit from a cognitive reappraisal exercise following mood induction (Diedrich, Hofmann, Cuijpers, & Berking, 2016). This study randomized participants to complete a negative mood induction followed by a preparatory acceptance induction, self‐compassion induction, or a waiting period, and then a period of cognitive reappraisal, with mood changes over the course of the experimental procedure as the outcome measure. Results indicated that self‐compassion, but not acceptance, significantly enhanced the effects of cognitive reappraisal compared to a waiting period. These studies focus on deliberate cultivation of self‐compassion in the service of emotion regulation, as part of an experimental procedure. However, their findings suggest that further research exploring the ways in which habitual cognitive emotion regulation strategy use and *dispositional* self‐compassion (i.e., participants' self‐reported tendencies toward self‐compassion in daily life) might interact with one another in depressed or at‐risk populations, or with interventions designed to support mood repair, is warranted.

### 1.1 Current studies

This paper reports two studies, one correlational and one experimental that together examine these issues in a group at risk for depression relapse/recurrence, on the basis of their clinical history of depressive episodes. We hypothesized that consistent with the findings of other recent work, dispositional self‐compassion would enhance the capacity to respond to negative events and negative mood in ways that lessen its secondary negative consequences (in this paper operationalized as continuing emotional dysregulation and self‐devaluation).

*Study 1* explored the relationship between dispositional self‐compassion (Self‐Compassion Scale [SCS]) and use of cognitive emotion regulation strategies such as positive reappraisal, positive planning, catastrophizing, and self‐blame, extending previous findings by focusing on a large sample of participants with a history of recurrent major depression (three or more prior episodes) and utilizing a measure which explores habitual use of cognitive and emotion regulation strategies.

*Study 2* examined the impact of individual differences in dispositional self‐compassion and reported use of positive and negative cognitive emotion regulation strategies on changes in mood and self‐devaluation across an experimental mood‐induction procedure. It considered the extent to which changes in mood and self‐devaluation differed as a function of experimentally induced response styles, with participants encouraged to engage in mindfulness, rumination or a period of silence during the mood repair phase. It also considered the interaction between dispositional tendencies (SCS and Cognitive Emotion Regulation Questionnaire [CERQ] scores) and induced response styles in determining changes in mood and self‐devaluation. Previous research suggests that rumination sustains negative mood (Nolen‐Hoeksema, Blair, & Lyubomirsky, 2008) and that mindfulness attenuates it (Keng, Tan, Eisenlohr‐Moul, & Smoski, 2017). However, it is unclear how dispositional factors interact with deliberate adoption of particular mood repair strategies to determine emotion regulation or indeed whether brief experimentally induced mood repair strategies have sufficient impact to override or modify trait‐like response tendencies. As a result, the examination of potential interaction effects between dispositional factors and induced mood repair strategies was exploratory. In summary, we aimed to answer the following research questions:

1. How are self‐compassion and cognitive emotion regulation strategy use associated in people with a history of recurrent depression (Study 1)?
2. Do individual differences in self‐compassion and cognitive emotion regulation strategy use in people at risk for depression determine levels of emotional disturbance and self‐devaluative processing following mood challenge? Does exploratory analysis suggest evidence that these relationships differ according to the type of mood repair participants are encouraged to employ (Study 2)?

## 2 STUDY 1: METHOD

### 2.1 Participants

Study 1 utilized baseline data from participants recruited to a published randomized controlled trial (Kuyken et al., 2015). Participants were recruited from urban and rural GP practices in the South West of England. Inclusion criteria for the trial were a diagnosis of recurrent major depressive disorder in full or partial remission, as assessed with the Structured Clinical Interview for DSM‐IV (SCID‐IV; Spitzer, Williams, Gibbon, & First, 1996) and aged 18 or older. Exclusion criteria were a current major depressive episode meeting full diagnostic criteria; co‐morbid diagnoses of current substance abuse; organic brain damage; current/past psychosis, including bipolar disorder; persistent antisocial behaviour and persistent self‐injury requiring clinical management/therapy. All participants were on a therapeutic dose of maintenance antidepressant medication. In total, 403 participants from the original trial sample had complete data on the SCS and the CERQ, with a further 21 participants from the original study excluded due to missing data. The overall sample comprised 306 women and 97 men with a mean age of 49·54 years (*SD* = 12.33). The sample was almost exclusively (99%) of Caucasian ethnicity. At the point of assessment, the mean level of depressive symptoms in the sample, as assessed by the Beck Depression Inventory Second Edition (BDI‐II; see below) was 14·09 (*SD* = 10.00), falling just within the range of mild depressive symptoms.

The study was granted ethical approval by the NHS Research Ethics Committee and by the School of Psychology Ethics Committee, University of Exeter.

### 2.2 Measures

#### 2.2.1 Structured Clinical Interview for DSM–IV (SCID‐IV)

The depression module of the SCID‐IV was used to establish a history of depression and current depression status (Spitzer et al., 1996). Clinical interviews were conducted by fully trained postgraduate research psychologists under supervision of a clinical psychologist (WK). All received training and established high rates of inter‐rater reliability (90% agreement, κ = 0.62, 95% CI [0.48–0.77], *p* < .0001 for within‐team double rating of first relapse or borderline relapse; 96% agreement κ = 0.90, 0.82–0.98, *p* < .0001 for independent re‐rating of subset of 112 SCID‐IV interviews).

#### 2.2.2 Self‐Compassion Scale (SCS)

The SCS is a 26‐item self‐report instrument, with each item rated on a 5‐point Likert scale (1 = *Almost Never* to 5 = *Almost Always*; Neff, 2003a). It yields a total score as well as scores on six subscales: self‐kindness, self‐judgement, common humanity, isolation, mindfulness, and over‐identification. Higher scores indicate higher levels for each respective scale, with reverse scoring of items loading onto the negatively framed subscales. Sample items include “I try to be loving towards myself when I'm feeling emotional pain” and “When times are really difficult, I tend to be tough on myself.” The SCS has good test–retest reliability (*r* = .93) and convergent and discriminant validity (Neff, Kirkpatrick & Rude, 2007). A recent psychometric evaluation broadly supports the subscales' reliability and validity in clinical samples (Neff, Whitaker, & Karl, 2017).

#### 2.2.3 Beck Depression Inventory Second Edition (BDI‐II)

The BDI‐II is 21‐item measure, with each item scored on a 4‐point scale, yielding a summary score ranging from 0 to 63. Scores of 0–13 are considered to reflect minimal symptoms of depression, 14–19 mild depression, 20–28 moderate symptoms of depression, and 29–62 severe symptoms of depression (Beck, Steer, & Brown, 1996). The measure has demonstrated excellent reliability, validity, and sensitivity to change (Beck, Steer, Ball, & Ranieri, 2010; Beck et al., 1996).

#### 2.2.4 Cognitive Emotion Regulation Questionnaire (CERQ)

The CERQ is a 36‐item self‐report measure of cognitive coping strategies used following a negative event or situation, with each item rated on a 5‐point scale (1 = [*Almost*] *never* to 5 = [*Almost*] *always*; Garnefski, Kraaij, & Spinhoven, 2002). It provides a total score as well as scores on nine subscales: self‐blame, acceptance, rumination, positive refocusing, refocus/planning, positive reappraisal, putting into perspective, catastrophizing, and other blame. Higher scores indicate higher levels for each respective scale (reverse scoring of some items is required for the total score). Sample items include “I think that I have to accept the situation” and “I am preoccupied with what I think and feel about what I have experienced.” The CERQ has demonstrated adequate reliability and validity (Garnefski & Kraaij, 2007). For the purposes of the current studies, we additionally summed the positive subscales (acceptance, positive refocusing, refocus/planning, positive reappraisal, and putting into perspective) and negative subscales (self‐blame, rumination, catastrophizing, and other blame) to produce two composite scores representing theoretically more and less adaptive cognitive coping responses, following Potthoff et al. (2016).

### 2.3 Statistical analysis

Data were analysed using statistical software SPSS version 22 (IBM Corp., 2013). Data were investigated for outlying values, skewness, and kurtosis. There were outlying values on each of the SCS subscales with the exception of common humanity as well as on the total score. On the CERQ, there were outlying values on the positive refocusing and other blame subscales. Kolmogorov–Smirnov tests indicated that none of the subscales were normally distributed. Because of the straightforward nature of the analyses, all cases were retained despite the presence of both some outlying values and non‐normality, and non‐parametric (Spearman's) correlation coefficients were computed to explore the associations between variables.

## 3 STUDY 1: RESULTS

### 3.1 Association between self‐compassion and emotion regulation

Table 1 shows the exploratory Spearman's correlation coefficients between the SCS total and facet scores and each of the CERQ subscales. There were significant positive correlations of moderate strength between the total SCS score and the CERQ subscales of positive refocusing, refocus/planning, positive reappraisal, and putting into perspective. Likewise, there were significant negative correlations between total SCS score and the CERQ subscales of self‐blame, other blame, rumination, and catastrophizing, which were very weak in strength for other blame, weak in strength for rumination, and moderate in strength for catastrophizing and self‐blame. There was no significant correlation between total SCS score and the CERQ subscale of acceptance. Summing across positive and negative CERQ subscales, there was a large positive correlation between total SCS score and positive CERQ subscales, *r* = 52, *p* < .001 and a large negative correlation between total SCS score and negative CERQ subscales, *r* = −.51, *p* < .001. All but three of the statistically significant correlation coefficients between SCS and CERQ facets (SCS self‐kindness and CERQ rumination, SCS self‐judgement and CERQ positive reappraisal, and SCS self‐judgement and CERQ other blame) remain significant following application of a Bonferroni correction to account for multiple comparisons and retain a familywise alpha of *p* < .005 (requiring individual correlation coefficients to *p* < .008).

**Table 1:** Correlations between Self‐Compassion Scale (SCS) and Cognitive Emotion Regulation (CERQ)

CERQ coping strategies									
Self‐compassion	Self‐blame	Acceptance	Rumination	Pos. Refocusing	Planning	Reappraisal	Perspective	Catastrophizing	Other blame
Self‐kindness	−.379**	.007	−.094	.475**	.452**	.471**	.400**	−.212**	−.028
Self‐judgement	−.518**	−.190**	−.395**	.217**	.172**	.171**	.111* ^,^ a	−.385**	−.130* ^,^ a
Common humanity	−.270**	.146*	−.029	.539**	.462**	.526**	.546**	−.226**	−.030
Isolation	−.465**	−.151*	−.370**	.221**	.210**	.207**	.220**	−.521**	−.280**
Mindfulness	−.255**	.150**	.008	.442**	.540**	.566**	. 520**	−.302**	−.089
Over‐identification	−.424**	−.040	−.392**	.268**	.223**	.251**	.282**	−.524**	−.259**
SCS total	−.502**	0.000	−.264**	.495**	.475**	.540**	.467**	−.479**	−.166*

a No longer statistically significant after Bonferroni correction to adjust for multiple comparisons (*p* < .008 to ensure familywise alpha <.05).

* Correlation is significant at the .05 level (2‐tailed).

** Correlation is significant at the .001 level (2‐tailed).

The above findings support previous work identifying associations between self‐compassion and positive cognitive restructuring (e.g., Allen & Leary, 2010) and extend these to a clinical sample with a history of recurrent depression. They suggest that self‐compassion is associated with the way that people respond to negative events and experiences. Study 2 moves beyond retrospective self‐report to bring the relationship between self‐compassion, cognitive emotional regulation strategy use, and responses to mood challenge under experimental control.

## 4 STUDY 2: INTRODUCTION

Study 2 was an experimental study designed to establish whether individual differences in self‐compassion and cognitive emotion regulation strategy use in people with a history of depression explained differences in mood and self‐devaluation following a period of mood induction and potential mood repair, in which each participant was invited to engage in one of three different mood repair strategies. The mood repair strategies to which participants were assigned were silence (intended to allow engagement in usual emotion regulation strategies), rumination (intended to encourage engagement in analytical self‐focused thinking), and mindful breathing (intended to encourage participants to decentre from thoughts and feelings and facilitate self‐compassionate responses).

## 5 STUDY 2: METHODS

### 5.1 Participants

Participants were drawn from individuals associated with the University of Exeter and the surrounding local community (recruited using posters), and people who had expressed an interest in participating in the PREVENT randomized controlled trial (described in methods of Study 1), but were ineligible because they had expereinced fewer than three previous depressive episodes. All participants had a history of depression in full or partial remission and scored <10 on the Hamilton Rating Scale for Depression (HAMD; J. B. W. Williams et al., 2008) to ensure that the experimental procedure did not exacerbate depressive symptoms in those currently experiencing significant residual symptoms (e.g., Kuyken, Byford, et al., 2010; Kuyken, Watkins, et al., 2010). Clinical interviews were conducted by fully trained postgraduate research psychologists under supervision of a clinical psychologist (WK). Participants received £10 payment for participation, or in the case of university students, given course credits.

### 5.2 Mood induction procedure

To assess responses to mood challenge, we employed the laboratory mood induction paradigm used by Segal et al. (2006) and described below. The task consisted of two parts, first, a negative mood induction and, second, a mood repair phase.

#### 5.2.1 Sad mood induction and manipulation check

To induce sad mood participants listened to sad music (Prokofiev's “Russia under the Mongolian Yoke” re‐mastered at half speed) for 8 min whilst rehearsing a sad memory. Participants were free to bring to mind any sad memory they chose and to rehearse this memory for the duration of the mood induction procedure. Sad mood was assessed premood and postmood induction using a visual analogue scale (VAS) from 0 (*I do not feel this way at all*) to 100 (*I feel this way very much or extremely*), and again following the mood repair phase, described below.

#### 5.2.2 Mood repair phase

Following the sad mood induction, participants were allocated by MW and JC to one of three conditions that were hypothesized to affect emotion regulation and, hence, degree of mood repair in a group at risk for depressive relapse. The groups were matched for age and gender and comparable on a range of other baseline measures (see later). Assignment was sequential, initially to the mindfulness and silence conditions with a third rumination condition added after study commencement. Both the mindfulness and rumination exercises were 4 min in duration and had identical opening instructions:

I am now going to play you an audio‐clip which lasts about 4 minutes. I would like you to sit in a comfortable upright position, close your eyes if you feel comfortable doing so or perhaps stare down at the floor, and follow closely the instructions on the CD. I will do the same thing so that we both do it together.

**Figure 1:**
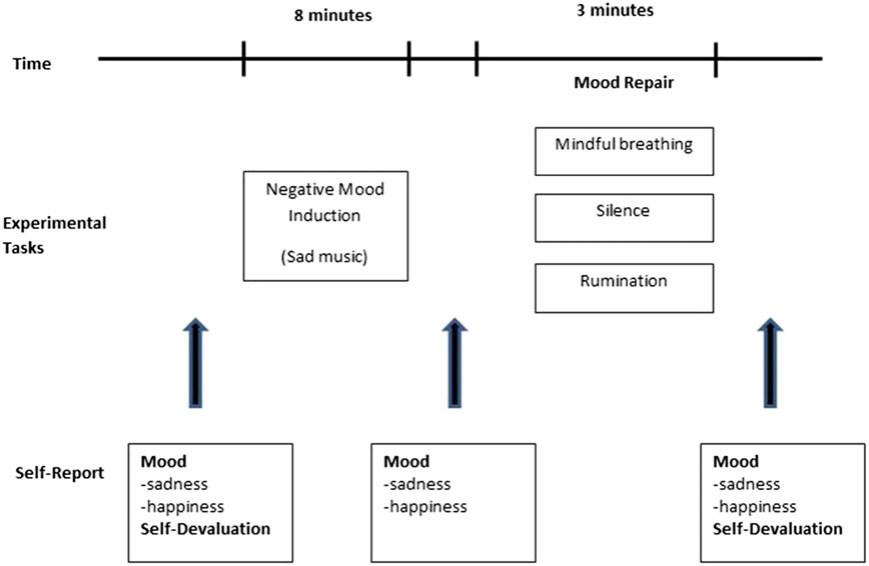
Experimental procedure in Study 2 [Colour figure can be viewed at http://wileyonlinelibrary.com]

In the mindfulness condition, participants were guided to (a) note their thoughts, feelings, and bodily sensations; (b) then firmly but kindly orient their attention to their breathing sensations, and (c) expand their awareness to moment‐by‐moment awareness of the whole body. In the rumination condition, participants were guided to consider the causes and consequences of sad mood. The silence condition was matched in length, but participants were instructed as follows: “I'd now like you to sit in silence for the next few minutes. I'll let you know when the time is up.” Figure 1 illustrates the experimental protocol.

### 5.3 Measures

#### 5.3.1 Study 1 measures

The CERQ, SCS, SCID‐IV, HAMD, and BDI‐II are described in the methods for Study 1.

Visual Analogue Scales of Mood were assessed using two 0–100 VAS scales at each time point, one for sadness and one for happiness, as shown in Figure 1. Participants were given the instruction to rate both sadness and happiness “at this moment.”

#### 5.3.2 Hamilton Depression Rating Scale (GRID HAMD)

The GRID‐HAMD is a manualized structured clinical interview, designed to assess 17 separate symptoms of depression (J. B. W. Williams et al., 2008). Each symptom is rated for severity on a 0–4 scale, based on participant responses to interviewer questions, and these scores are summed to yield a total score. The GRID‐HAMD is a revision of the earlier Hamilton Depression Rating Scale and allows for differentiation of symptom intensity and frequency, whilst producing scores that map onto those derived from earlier versions of the HAMD. The GRID‐HAMD has good inter‐rater reliability.

#### 5.3.3 Depressed States Checklist (DSC)

The DSC assesses endorsement of self‐devaluative adjectives thought to be activated at times of low mood in people at risk for depressive relapse (e.g., “abandoned,” “a failure,” and “pathetic”; Teasdale & Cox, 2001. Respondents indicate how far they endorsed these adjectives when their mood started to go down in the last month on a 4‐point scale (0 = *Not at all* to 3 = *Very or extremely*). The measure shows good reliability and discriminant validity. We analysed a sum‐score corresponding to endorsement of the 14 adjectives that are indicative of self‐devaluation (following Ehring, Ehlers, & Glucksman, 2008). Although not ostensibly a state measure, we hypothesized that reinstatement of depressogenic thinking following mood induction would lead to increased endorsement of self‐devaluative adjectives and, thus, indicate increased self‐devaluation at a time of low mood.

### 5.4 Procedure

Participants were initially screened via telephone to assess eligibility. Eligible participants were mailed the study information sheet and questionnaire booklet including the CERQ, SCS, and BDI‐II to complete and send back/bring with them to the testing session. Following informed consent, the study procedure followed the flow depicted in Figure 1. Participants first completed the DSC, were assessed for eligibility using the SCID‐IV and HAMD, and then completed the first VAS ratings (T1). All participants then completed the mood induction paradigm, followed by a further set of VAS ratings (T2). Participants then followed the instructions for their assigned mood repair condition. Finally, all participants completed the DSC again alongside a final set of VAS ratings (T3). At the end of the study, participants were debriefed and offered some exercises to repair mood if necessary.

### 5.5 Statistical analysis

Data were analysed using statistical software SPSS version 22 (IBM Corp., 2013). Data were inspected for prerequisitions for the general linear model (i.e., normal distribution, variance homogeneity, sphericity for mixed/repeated measures ANOVAs and normality of residuals, multicollinearity for multiple regression). No participants were excluded but four had missing data on the BDI‐II and were thus omitted from some analyses.

#### 5.5.1 Mood induction

To test the impact of mood induction and mood repair on participant's mood and DSC score, a series of repeated measures ANOVAs with time (T1, T2, T3 for mood, T1 and T3 for DSC) as within‐subjects factor and condition (silence, mindful breathing, and rumination) as between‐subjects factor were conducted. Main effects and interactions were followed up by Bonferroni‐corrected post hoc test and simple contrasts.

#### 5.5.2 Correlational analyses

In order to examine the association between individual differences in SCS and CERQ scores and change in mood and DSC scores in individuals assigned to each of the mood repair conditions (mindful breathing, silence, rumination), zero order correlations and a series of stepwise linear regressions were calculated with the residualized gain scores as outcome and with dummy‐coded condition, SCS, CERQ, and BDI‐II as predictors. Mindful breathing and rumination were entered as dummy‐coded variables with the silence condition being the reference category. Residualized gain scores, a validated index of pre–post change which controls for variance in initial pre‐scores, were calculated as the difference between the actual postrepair score and the expected postrepair score (calculated by regression of raw post‐score on pre‐score; Hofmann, 2004; Speckens, Ehlers, Hackmann, & Clark, 2006; Steketee & Chambless, 1992). In addition, a series of moderation analyses following procedures by Aiken and West (1991) were performed using mean‐centred continuous predictors and interaction terms of condition and trait as predictors.

## 6 STUDY 2: RESULTS

### 6.1 Participant characteristics

The sample comprised 68 participants, 46 women (68%) and 22 men (32%), with a mean age of 30 years (*SD* = 14.81, range 18–76) and was predominantly Caucasian (96%). The majority were students (63%) with the remainder employed (23%), retired (9%), or unemployed/homemakers/long‐term sick (5%). All had a history of major depression, with 39% reporting three or more episodes. Mean BDI‐II score at the time of participation was 7.59 (*SD* = 6.88, range 0–24). The three mood repair groups did not differ in gender (*rumination*: 6 male, 16 female; *mindfulness*: 8 male, 15 female; *silence*: 8 male, 15 female), age, *F*(2, 65) = 1.11, *p* = .34, BDI‐II score, *F*(2, 61) = .93, *p* = .40, SCS score, *F*(2, 65) = .723, *p* = .49 or the CERQ negative, *F*(2, 65) = 1.03, *p* = .36 or negative, *F*(2, 65) = .789, *p* = .46.

### 6.2 Mood change following mood induction

#### 6.2.1 Sadness

**Figure 2:**
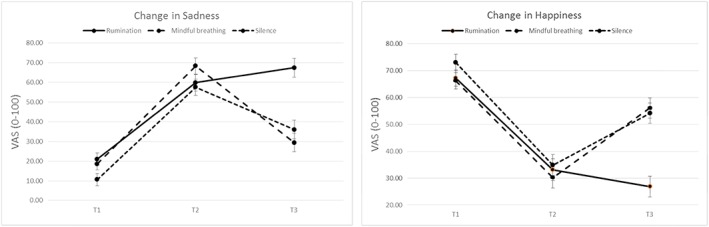
Self‐reported sadness and happiness in the experimental groups at premood induction (T1), postmood induction (T2), and postmood repair (T3) [Colour figure can be viewed at http://wileyonlinelibrary.com]

Repeated measures ANOVA with Time as within‐subjects factor and Condition as between‐subjects factor revealed significant main effects of Time, *F*(2, 64) = 145.46 *p* < .001; η_p_^2^ = .827, and Condition, *F(*1, 65) = 7.65; *p =* .001; η_p_^2^ = .190, and a significant Time × Condition interaction, *F*(4, 130) = 7.22; *p* < .001; η_p_^2^ = .182. Post hoc tests of the main effect of time (simple contrasts) revealed that sadness ratings differed significantly between time points, with significantly increased sadness at T2, *F*(1, 65) = 292.22; *p =* .001; η_p_^2^ = .818, and T3, *F*(1, 65) = 75.80; *p =* .001; η_p_^2^ = .538, as compared to T1. Following up the Time × Condition interaction, it was shown that at T3 individuals in the rumination condition reported significantly higher sadness than those in the silence (mean difference = 31.41, *p* < .001, CI [14.84, 47.99] and mindful breathing condition (mean difference = 38.06, *p* < .001, CI [21.49, 54.64]. In the rumination condition, reported sadness at T3 was significantly higher than at T1, *F*(1, 21) = 64.85; *p* < .001; η_p_^2^ = .755, but did not significantly differ from T2, *F*(1, 21) = 2.39; *p =* .137; η_p_^2^ = .102. In the mindful breathing condition, reported sadness at T3 was significantly lower than at T2, *F*(1, 22) = 64.85; *p* < .001; η_p_^2^ = .714, and tended to be higher than at T1, *F*(1, 22) = 3.24; *p =* .086, η_p_^2^ = .128. In the silence condition, reported sadness at T3 was significantly higher than at T1, *F*(1, 22) = 30.47; *p* < .001; η_p_^2^ = .581, and significantly lower than at T2, *F*(1, 22) = 12.05; *p =* .002, η_p_^2^ = .354. This indicates that (a) the sad mood induction was successful across conditions to induce sad mood and that (b) individuals in the silence and mindful breathing conditions show reductions in sad mood between T2 and T3 (due to dissipation of negative mood or mood repair) whereas those in the rumination condition do not (see Figure 2).

#### 6.2.2 Happiness

Repeated measures ANOVA yielded significant main effects of Time, *F*(2, 64) = 152.60 *p* < .001; η_p_^2^ = .827, and Condition, *F*(1, 65) = 4.19; *p =* .019; η_p_^2^ = .114, and a significant Time × Condition interaction, *F*(4, 130) = 9.30; *p* < .001; η_p_^2^ = .223. Post hoc tests of the main effect of time (simple contrasts) revealed that happiness ratings differed significantly between time points with significantly reduced happiness at T2, *F*(1, 65) = 270.08; *p =* .001; η_p_^2^ = .806, and T3, *F*(1, 65) = 139.93; *p =* .001; η_p_^2^ = .683, as compared to T1. Following up the interaction, it was shown that at T3 individuals in the rumination condition reported significantly lower happiness than those in the silence (mean difference = −27.31, *p* < .001, CI [−40.60, −14.03]) and mindful breathing condition (mean difference = −29.27, *p* < .001, CI [−42.55, −15.98]). In the rumination condition, reported happiness at T3 was significantly lower than at T1, *F*(1, 21) = 137.73; *p* < .001; η_p_^2^ = .868, but did not significantly differ from T2, *F*(1, 21) = 2.13; *p =* .159; η_p_^2^ = .092. In the mindful breathing condition, reported happiness at T3 was significantly lower than at T1, *F*(1, 22) = 7.69; *p =* .011; η_p_^2^ = .259, and significantly higher than at T2, *F*(1, 22) = 40.86; *p* < .002, η_p_^2^ = .650. In the silence condition, reported happiness at T3 was significantly lower than at T1, *F*(1, 22) = 38.26; *p* < .001; η_p_^2^ = .635, and significantly higher than at T2, *F*(1, 22) = 24.54; *p* < .001, η_p_^2^ = .527. At T1 and T2 groups did not differ from each other significantly. This indicates that (a) the sad mood induction was successful across conditions in reducing happy mood and (b) that individuals in the silence and mindful breathing conditions show significant improvements in their happiness between T2 and T3 whereas those in the rumination condition did not (see Figure 2).

### 6.3 Self‐devaluation (DSC) following mood induction

**Figure 3:**
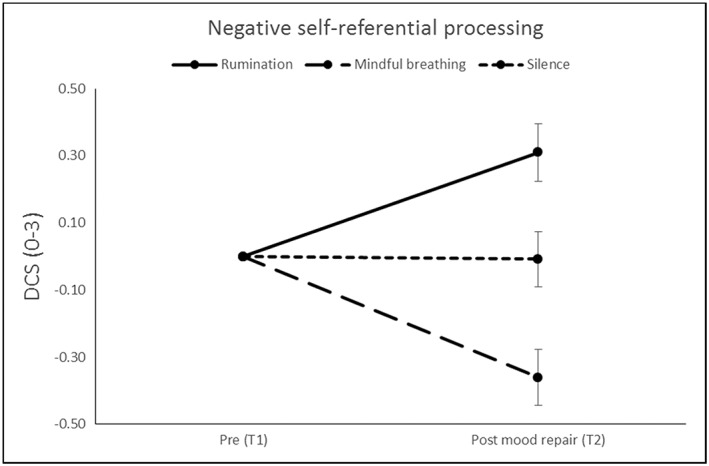
Self‐devaluation assessed via the Depressed States Checklist (DSC), in the experimental groups at premood induction (T1, baseline corrected) and postmood repair (T3)

To examine the effects of the mood induction and mood repair phases on DSC score a repeated measures ANOVA with time as within‐subjects factor and condition as between‐subjects factor was conducted. This revealed a significant Time × Condition interaction, *F*(2, 65) = 15.89; *p* < .001; η_p_^2^ = .328, but no main effects of time or condition. Post hoc tests showed significant pre (T1)–post (T3) increase in DSC score in the rumination condition, *F*(1, 21) = 11.55; *p =* .003; η_p_^2^ = .355, and significant pre (T1)–post (T3) decrease in DSC score in the mindful breathing condition, *F*(1, 22) = 23.03; *p* < .001; η_p_^2^ = .511. There was no significant effect of time in the silence condition, *F*(1, 22) = 0.12; *p =* .913; η_p_^2^ = .001. In addition, post‐experiment, individuals in the rumination condition showed significantly higher DSC score as compared to the other two groups collapsed, *F*(1, 67) = 4.96; *p =* .029. Changes in DSC score from premood induction to postmood repair are shown in Figure 3.

### 6.4 Self‐compassion, cognitive emotion regulation strategy use and responses to different forms of mood repair

Table 2 shows the zero order correlations between residualized gain scores for sadness, happiness (T2 to T3) and DSC score (T1 to T3), condition (rumination, mindful breathing, and silence); and BDI‐II, SCS, and positive and negative CERQ.

**Table 2:** Zero order correlations between residualized gain scores for sadness, happiness, and self‐devaluation and measures of depression (BDI‐II), self‐compassion (SCS), and cognitive emotion regulation (CERQ subscales)

	Variables	1	2	3	4	5	6	7	8	9
1	RGS_sadness									
2	RGS_happiness	−.654**								
3	RGS_self‐devaluation	.282*	−.252*							
4	Rumination	.344**	−.356**	.124						
5	Breathing	−.099	.141	.044	−.494**					
6	Silence	−.242*	.211	−.166	−.494**	−.511**				
7	SCS total	−.370**	.293*	−.566**	−.126	−.004	.129			
8	CERQ neg total	.214	−.057	.439**	.172	−.055	−.114	−.464**		
9	CERQ pos total	−.257*	.115	−.308*	−.126	.139	−.014	.539**	−.273*	
10	BDI total score	.367**	−.269*	.522**	.144	.011	−.155	−.582**	.302*	−.351**

* Correlation is significant at the .05 level (2‐tailed).

** Correlation is significant at the .01 level (2‐tailed).

#### 6.4.1 Change in sadness

In order to determine what predicts change in sadness during the mood‐repair phase, a stepwise multiple linear regression was run with residualized gain score for sadness pre (T2)–post (T3) mood repair as outcome/dependent variable and mood‐repair condition, total SCS, positive CERQ, negative CERQ, and BDI‐II (all assessed at T1) as predictor/independent variables. Prerequisitions for regressions (see details under methods) were met. The overall model was significant, *F*(2, 67) = 9.53, *p* < .001 and explained 23% variance (*R* ^2^ = .227). Only condition and SCS made a significant contribution; being in the rumination condition (β *=* .302, *t* = 2.75, *p =* .008) as opposed to mindful breathing (β *=* .066, *t* = 0.52, *p =* .604) was associated with greater increases in sadness whereas having a higher SCS score was associated with lower increases in sadness, β *=* −.332, *t* = −3.02, *p* = .004). Neither positive (β *=* −.057, *t* = −0.44, *p =* .663), negative CERQ (β *=* .011, *t* = 0.09, *p =* .930), nor BDI (β *=* .181, *t* = 1.44, *p =* .154) significantly explained variance. In a subsequent moderation analysis, we entered rumination, SCS score and an interaction term into a multiple linear regression which revealed main effects for the rumination condition (β *=* .322, *t* = 2.66, *p =* .010) and SCS (β *=* −.355, *t* = 2.749, *p =* .005) but no effect for the interaction term (β *=* −.066, *t* = −0.54, *p =* .589). This suggests that, in the presence of SCS score and its interaction with condition, increases in sadness during mood repair are associated with being in the rumination condition and having lower SCS scores. Both factors made an independent contribution to explaining variance and the interaction term only explained small levels of variance resulting in no significant moderation effect of SCS on the association between condition and sadness.

#### 6.4.2 Change in happiness

IThe stepwise regression to determine what predicts change in happiness during the mood repair (T2 to T3) was run with residualized gain score for happiness pre (T2)–post (T3) mood repair task as outcome/dependent variable and condition, total SCS, positive CERQ, negative CERQ, and BDI as predictor/independent variable. Prerequisitions for regressions (see details under methods) were met. The overall model was significant, *F*(2, 67) = 7.59, *p =* .001 and explained 19% variance (*R* ^2^ = .189). Only condition and SCS made a significant contribution; again, being in the rumination condition (β *=* −.324, *t* = 2.88, *p =* .005) rather than the mindfulness (β *=* −.025, *t* = 0.19, *p =* .850) condition was associated with greater decreases in happiness, whereas having higher SCS score was associated with greater increases in happiness (β *=* .253, *t* = 2.24, *p =* .028). Neither positive (β *=* −.087, *t* = −0.65, *p =* .516), negative CERQ (β *=* .150, *t* = 1.19, *p =* .240), nor BDI (β *=* −.147, *t* = 1.13, *p =* .262) significantly explained variance. In a subsequent moderation analysis, we entered rumination, SCS score and an interaction term into a multiple linear regression which revealed main effects for condition (β *=* −.339, *t* = −2.79, *p =* .007) and SCS (β *=* .268, *t* = 2.20, *p =* .031) but no effect for the interaction term (β *=* .060, *t* = 0.46, *p =* .648).

**Figure 4:**
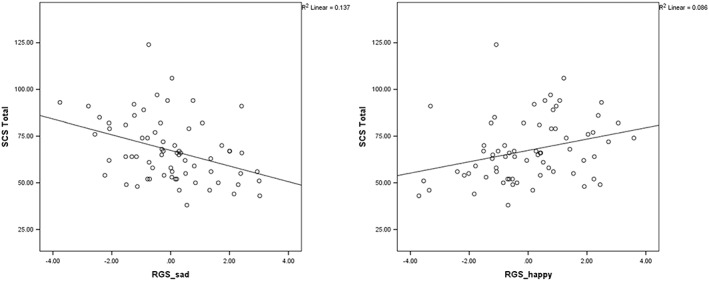
Correlations between self‐compassion and mood change (residualized gain scores) during mood repair task

This suggests that decreases in happiness during mood repair are driven by being in the rumination condition and having lower levels of self‐compassion but both factors made an independent contribution to explaining variance and the interaction term explained only small levels of variance indicating no significant moderation effect of self‐compassion on the association between condition and happiness. Scatterplots showing the association between mood change and SCS score are shown in Figure 4.

### 6.5 Self‐compassion, cognitive emotion regulation strategy use and self‐devaluation following mood induction and mood repair

In order to determine what predicts change in DSC score from premood induction to postmood repair, a stepwise multiple linear regression was run with residualized gain score for DSC as outcome/dependent variable and condition, change in mood (residualized gain scores), total SCS, positive CERQ, negative CERQ, and BDI as predictor/independent variables. Prerequisitions for regressions (see details under methods) were met. The overall model was significant, *F*(2, 67) = 18.32, *p* < .001 and explained 36% variance (*R* ^2^ = .361). Only SCS and negative CERQ made a significant contribution; smaller increases in self‐devaluation from pre to post, the experimental procedure were associated with higher SCS (β *=* −.462, *t* = −4.126, *p* < .001) and lower negative CERQ (β *=* .225, *t* = −2.010, *p =* .049). Neither self‐reported mood change (sad: β *=* .073, *t* = 0.682, *p* = .498; happy: β *=* −.115, *t* = −1.102, *p* = .275), experimental condition (mindfulness: β *=* .055, *t* = 0.549, *p* = .585; rumination: β *=* .028, *t* = 0.274, *p* = .785), nor positive CERQ (β *=* .003, *t* = 0.024, *p* = .981) or BDI (β *=* .200, *t* = 1.775, *p* = .081) made a significant contribution.

In a subsequent moderation analysis, we entered rumination, SCS and an interaction term into a multiple linear regression which revealed only a main effect for SCS (β *=* −.597, *t* = −4.91, *p =* .001) but no significant effects for rumination (β *=* .061, *t* = 0.49, *p =* .624) or for the interaction term (β *=* −.019, *t* = −0.16, *p =* .875). Similar effects were found for the moderation analyses using the negative CERQ. Here, there was a main effect for negative CERQ (β *=* .417, t = −4.91, *p =* .001) but no significant effects for rumination (β *=* .056, *t* = 0.49, *p =* .624) or for the interaction term (β *=* −.020, *t* = −0.16, *p =* .875). Similar non‐significant moderation effects were revealed when mindful breathing, SCS and an interaction term were entered.

These findings suggest that changes in self‐devaluation from T1 to T3 are explained by individual differences in self‐compassion and negative emotion regulation, rather than mood repair condition.

## 7 DISCUSSION

Sad mood is a normal, adaptive emotion. However, in those at risk for recurrent depression, it has developed unhelpful associations with self‐devaluative beliefs and maladaptive emotion regulation strategies such as self‐blame, rumination, and catastrophizing. The studies presented in this paper build on previous research which has suggested that self‐compassion may be important in helping people at risk for depressive relapse/recurrence manage low mood and the activation of negative mental content and process that often accompanies it, in an adaptive way (Krieger et al., 2016; Neff, 2003a, 2003b; Pauley & McPherson, 2010). The findings of Study 1 extend previous research on the association between self‐compassion and adaptive emotional regulation (Krieger et al., 2015; Leary et al., 2007) to a clinical sample with a history of recurrent depression, demonstrating that in this population dispositional self‐compassion, assessed by the SCS, was associated with greater endorsement with adaptive cognitive emotion regulation strategy use. Likewise, the identification of associations between the SCS and maladaptive coping responses such as self‐blame and catastrophizing are also consistent with earlier research (Raes, 2010). Interestingly, the only exception to the general pattern of results was for the CERQ acceptance subscale. Higher scores on this subscale were associated, albeit weakly, with participants' reporting of both greater mindfulness and common humanity and also greater isolation and self‐judgement. These findings, which appear counter‐intuitive, may reflect the item content on the CERQ acceptance subscale. This subscale contains items which reflect both the non‐judgemental acceptance characteristic of a more mindful orientation to experience, for example, “I think that I have to accept that this has happened” and “I think that I have to accept the situation”, but also items that have a tone more characteristic of resignation or defeat “I think I cannot change anything about it” and “I think I must learn to live with it.” This ambiguity in item content may explain the unexpected lack of association of the CERQ acceptance subscale with self‐compassion, despite the theoretical overlap between these constructs. Overall, the results suggest that individual differences in self‐compassion and its constituent facets appear to be meaningfully related to the strategies that people with a history of depression report employing to cope with difficult or stressful situations.

Study 2 examined the association between dispositional self‐compassion, use of cognitive emotion regulation strategies, and changes in mood and self‐devaluation in participants exposed to a negative mood induction followed by mood repair (mindfulness, rumination, silence). The findings point to a significant role for individual differences in self compassion and negative cognitive emotion regulation strategy use in influencing responses to a mood challenge procedure among people at risk for depressive relapse/recurrence, both in terms of mood recovery (SCS) and changes in self‐devaluation (SCS and negative CERQ). These findings suggest that self‐compassion signifies a general ability to tolerate and regulate intense emotions, a capacity which is likely to be important to resilience and wellbeing in a highly vulnerable group.

Instructing individuals to ruminate following mood challenge sustained negative mood, and instructing them to engage in a period of silence or mindfulness was associated with reductions in negative mood. However, exploratory analyses suggested that the effects of SCS on mood and self‐devaluation, and negative CERQ on self‐devaluation, were similar irrespective of how an individual was instructed to relate to their sad mood. The absence of statistically significant interactions between participants' levels of self‐compassion or cognitive emotion regulation and the effects of the different mood repair strategies can possibly be explained by the small effect size and the sample size (e.g., Alexander & DeShon, 1994). The sample required to render an interaction effect of the size obtained in this study (e.g., *R* ^2^ = .02) statistically significant would need to be much larger (e.g., *N* = 395) which would have made our experimental study unfeasible. It would therefore be premature to conclude that the association between type of mood regulation and reported mood or self‐devaluation is not moderated by dispositional self‐compassion or emotion regulation and future large scale multicentric replications should test this assumption.

### 7.1 Limitations

The studies reported in this paper should be interpreted in the light of a number of factors. First, there is a need to consider the best way to interpret the effects of the different mood repair strategies on persistence of negative mood. One possibility is that rather than the mindfulness and silence conditions producing active mood repair, reductions in negative mood in these two conditions may simply reflect natural recovery. However, the mindfulness condition was associated with significant reductions in self‐devaluation, whereas in the silence condition there was no significant change. This suggests some differential action of the two mood repair strategies. One possibility is that instructions to engage in a mindful breathing practice may provide distraction from self‐devaluative thoughts, or through the emphasis on acceptance of mind wandering and sustained attention on the breath, may have limited their proliferation and persistence. Future work which includes a distraction condition as well as mindfulness, rumination, and silence conditions would help to address this issue.

A second issue concerns the fact that in an ideal study design, self‐devaluation would have been measured on three occasions; prior to the mood induction and both prior to and following the mood repair phase. Concerns about the impact of multiple repeated administration of the DSC meant that it was only included at the first and third assessment points. However, since dispositional self‐compassion may influence both the extent to which mood induction activates self‐devaluative cognitions and the extent to which such cognitions diminish during mood repair, a design which allowed these two effects to be separated would have been preferable and would be an interesting topic for future research.

A third issue concerns the nature of the sample in Study 2, which was composed of individuals with a history of depression but low levels of residual symptoms. Our findings therefore illuminate cognitive processes in those at risk of depressive relapse/recurrence rather than those at risk of a first episode of depression. Models of cognitive reactivity suggest that habitual response patterns are established across depressive episodes and, particularly, in the case of self‐devaluation, it is likely that it is these habitual response patterns that are observed. Thus, it is not clear to what extent our findings would also generalize to the relationship between self‐compassion and responses to mood induction in those who had not yet experienced a depressive episode but were vulnerable. Likewise, by excluding those with high levels of residual symptoms (for ethical reasons, in a design with negative mood induction), we may have excluded those most likely to show cognitive reactivity in response to the mood induction paradigm, thus attenuating effects. Our sample also contained a majority of female participants, and so it is unclear whether the findings would generalize to majority male samples.

Finally, random assignment was not used in Study 2. Thus, whilst the three experimental groups did not differ in age, gender, baseline residual depressive symptoms, baseline CERQ scores, or baseline SCS, it is possible that there may have been undetected differences between the populations and a fully randomized design would have strengthened the findings.

## 8 CONCLUSIONS

Although self‐compassion can be conceptualized as an individual difference variable, it is also a capacity that can be enhanced through a range of psychological therapies (e.g., Gilbert, 2009; Kabat‐Zinn, 2013; Segal, Williams, & Teasdale, 2013). There is evidence that development of self‐compassion is one crucial aspect of the change process in mindfulness‐based stress reduction (Shapiro, Brown, & Biegel, 2007) and mindfulness‐based cognitive therapy (Kuyken, Watkins, et al., 2010); and studies suggest that in interventions that train self‐compassion, such training is associated with a range of positive outcomes (Hofmann, Grossman, & Hinton, 2011; Kuyken, Byford, et al., 2010; Kuyken, Watkins, et al., 2010). Our findings support the idea that self‐compassion supports adaptive responses to mood challenge including more rapid mood recovery, greater engagement in positive, proactive problem solving strategies, and less engagement in maladaptive responses to negative experiences. It would be advantageous for future work to examine whether such associations translate into therapeutic contexts in which self‐compassion is deliberately enhanced, either explicitly or implicitly, and thus the extent to which engagement in positive coping strategies accounts for some of the beneficial effects of such approaches on mental health outcomes.

## AUTHOR CONTRIBUTIONS

The study was designed by WK, MJW, and AK. MJW and JC collected data for Studies 1 and 2. AK conducted the statistical analyses and CC drafted the manuscript. All authors contributed to data interpretation, commented on the draft manuscript, and revised it for important intellectual content. The PREVENT trial that contributed data for Study 1 is described in Kuyken et al., 2015.

## CONFLICT OF INTERESTS

WK is the director of the Oxford Mindfulness Centre. WK receives payments for training workshops and presentations related to MBCT and donates all such payments to the Oxford Mindfulness Foundation, a charitable trust that supports the work of the Oxford Mindfulness Centre. WK was until 2015 an unpaid Director of the Mindfulness Network Community Interest Company and gave evidence to the UK Mindfulness All Party Parliamentary Group. CC is affiliated with the Oxford Mindfulness Centre but does not receive additional remuneration for training workshops or presentations related to MBCT.

## FUNDING INFORMATION

The funders had no role in the study design, in the collection, analysis and interpretation of the data, in the writing of the report, and in the decision to submit the article for publication.
